# Effect of Sonic Hedgehog on the Regeneration of Epidermal Texture Patterns

**DOI:** 10.3390/biomedicines10123099

**Published:** 2022-12-01

**Authors:** Kento Takaya, Noriko Aramaki-Hattori, Shigeki Sakai, Keisuke Okabe, Kazuo Kishi

**Affiliations:** Department of Plastic and Reconstructive Surgery, Keio University School of Medicine, Shinjuku, Tokyo 160-8582, Japan

**Keywords:** sonic hedgehog, skin texture, scar, mouse fetus, regeneration

## Abstract

Wounds on embryonic mouse fetuses regenerate up to embryonic day (E) 13, but after E14, the pattern is lost and a visible scar remains. We hypothesized that the sonic hedgehog (Shh), which is involved in patterning during development, is involved in the regeneration of texture. Embryos of ICR mice were surgically injured at E13, E14, and E15 and analyzed for the expression of Shh. For external Shh administration, recombinant Shh-containing slow-release beads were implanted in the wounds of mice. In contrast, cyclopamine was administered to wounds of adult mice to inhibit Shh. The expression of Shh was unaltered at E13, whereas it was upregulated in the epidermis of the wound from E14 onward. Implantation of recombinant Shh-containing beads into E13 wounds inhibited skin texture regeneration. Cyclopamine treatment inhibited epithelialization and thickening of the epidermis in the wounds of adult mice. In vitro, Shh promoted proliferation and inhibited the migration of epidermal keratinocytes through the activation of cyclin D proteins. Thus, our results suggested that the expression of Shh is involved in the regeneration of texture during wound healing, especially in epidermal keratinocyte migration and division, and could inhibit skin texture regeneration after E14.

## 1. Introduction

Fetal skin wounds made at certain developmental stages regenerate completely without scarring. For example, wounds inflicted on mouse fetuses on gestational (E) days 13–16 are completely histologically healed [1]. Hair follicles and sebaceous glands are regenerated and the dermal extracellular matrix is deposited in a manner similar to that in uninjured skin. However, macroscopic observation of healed wounds after E14 reveals a slightly visible mark at the surgical site despite histological regeneration, a discrepancy observed in previous studies using *Monodelphis domestica* [2]. One of the main reasons for the appearance of this mark is that the pattern of furrows, papillae, and fine wrinkles of the epidermis, that is, the texture, disappears or is disrupted on the scar [3].

Complete skin regeneration requires the restoration of the surface structure, as of well as the dermis and skin appendages. Therefore, it is necessary to delineate the difference in the wound-healing process after E13 and E14, which is the switch between the presence and absence of a visible scar on the skin. The interaction between the epidermis and the dermis was reported to be maintained at E13, which could regenerate texture [1]. The regeneration of the complex structure of the skin texture could involve epidermal-dermal interactions and patterning factors during wound healing.

One factor involved in patterning is hedgehog protein. Hedgehog, an important determinant of cell fate, is involved in the patterning of the skeleton and organs during the development of various organisms [4,5]. Sonic hedgehog (Shh) is expressed in the ectoderm to form hair follicles and feathers during normal development and has been recently shown to play an important role in hair development [6]. Coculture experiments of the epidermal and dermal components of embryonic skin revealed that epidermal development and postnatal hair follicle formation were regulated by a reciprocal beneficial interaction between the epithelium (epidermis) and the mesenchyme (dermis) [7]. As it has been long noted that hairs grow at skin texture intersection sites during mammalian development [8], this suggested the involvement of Shh and skin texture regeneration in determining hair follicle patterning.

A recent study on Shh and wounds reported that the regeneration of new hair follicles during the healing of major skin excisions in mice involves Shh signaling that plays a major role in the conversion of wound fibroblasts from scar-promoting to hair follicle neogenesis stimulated fibroblasts. This phenomenon can be referred to as true tissue regeneration [9,10,11].

This link between Shh and hair follicle regeneration, a true tissue regeneration, has attracted widespread interest, but its relevance to complete skin regeneration, including texture, remains unclear.

We hypothesized that Shh can determine epidermal patterning and could be involved in the transition from complete regeneration of epidermal patterns in E13 wound healing to visible scar formation in late fetal wound healing.

We analyze Shh expression of Shh in the epidermis during fetal wound healing and investigated the effect of Shh on the formation of skin furrows and skin papules. We focused on the 12- and 24 h post-injury timepoints of the wound-healing process, as fetal mouse wounds heal quickly, completing epithelialization in approximately 48–72 h [1]. We also investigated the role of Shh in the regeneration of chymes during wound healing by suppressing its expression using cyclopamine, an inhibitor of Shh, and inducing wound-specific stimulation of Shh using slow-release beads.

## 2. Materials and Methods

### 2.1. Ethical Considerations

The research protocol was reviewed and approved by the Institutional Animal Care and Use Committee of the Keio University School of Medicine (approval number: 20170914). All experiments were conducted according to the institutional guidelines for animal experiments at the Keio University. This study was reported in accordance with the Reporting of In Vivo Experiments on Animals (ARRIVE) guidelines.

### 2.2. Embryonic Wounding and Skin Harvesting

Eight-week-old ICR mice were obtained from Sankyo Laboratories (Tokyo, Japan). Embryos with a vaginal plug observed in the maternal vagina were designated E0. Surgery was performed on E13, E14, and E15 fetuses. Pregnant maternal-fetal mice were anesthetized with 4% isoflurane (FUJIFILM Wako Pure Chemical Co., Ltd., Osaka, Japan), which was maintained at 2% during surgery. The surgical site was sterilized with 70% ethanol, the myometrium and amnion were opened, and a 2-mm-long full-layer incision was made in both flanks of the fetus using sterile microsurgical scissors (Medical U&A, Inc., Osaka, Japan) (n = 12 per timepoint). In E13 and E14, after wound creation, only the amniotic and yolk sacs were sutured with 9-0 nylon threads, whereas the myometrium was left open and unstitched. In E1, after wounding the fetus, only the myometrium was sutured with 9-0 nylon threads, the uterus was returned to the abdominal cavity and the abdomen was closed. Before closing the abdomen, 1 μg ritodrine hydrochloride (uterine relaxant; FUJIFILM Wako Pure Chemical Co., Ltd. per g of body weight was administered intraperitoneally and the peritoneum and skin of the body were continuously sutured with 5-0 nylon threads. Four fetuses per mother mouse were operated on. At various timepoints after injury, the animals were sacrificed by cervical dislocation after anesthesia with 4% isoflurane inhalation. Skin from the wounded fetuses was collected 12 and 24 h after injury. The intact skin was used as a control and to evaluate the Shh expression of Shh during normal skin development. Tissue specimens were fixed by immersion in 4% paraformaldehyde, embedded in paraffin, and maintained at 23 ± 2–3 °C (room temperature) until sectioning.

### 2.3. Adult Mouse Wounding

Ten-week-old male ICR mice were anesthetized with isoflurane and two full-layer wounds (approximately 1 cm in diameter) were created per mouse on both sides of the chest using a No. 11 scalpel on the shaved back skin. This procedure was performed on five adult mice. At 0, 12, 24, 72, and 120 h after wounding, the mice were euthanized by cervical dislocation. The wounds were then excised, harvested and fixed overnight in 4% paraformaldehyde, soaked in 20% sucrose/phosphate-buffered saline (PBS), embedded in OCT compound (Sakura Finetek Japan Co., Ltd., Tokyo, Japan) and cut into 7-μm–thick sections.

### 2.4. Gel Beads Transplantation in E13 Fetuses

Mouse recombinant Shh (R&D Systems, Inc., MN, USA) was dissolved in PBS to make a 1 µg/µL Shh solution. Cross-linked agarose beads (Aff-gel blue beads, Bio-Rad Laboratories, Inc., CA, USA) were immersed in PBS for 24 h to prepare Shh-soaked beads. The control beads were soaked in bovine serum albumin (1 µg/µL) dissolved in PBS and placed in the wound of E13 fetuses. The fetuses were collected after 72 h, Indian ink was dripped onto the skin, and the texture was observed under a stereomicroscope.

### 2.5. Shh Suppression by Cyclopamine in Adult Mouse Wound Healing

Ten-week-old male ICR mice were anesthetized with isoflurane inhalation. The wounds were then treated with 100 µL drops of cyclopamine (LKT Laboratories, Inc., St. Paul, MN, USA) at various concentrations (low, 500 ng/µL or high, 50 µg/µL) dissolved in PBS and protected with a clear film once daily. The control wounds were treated with 100 µL of PBS dropwise. After 7 d, the animals were euthanized by cervical dislocation and the wounds were excised and collected. The tissues were fixed by immersion in 4% paraformaldehyde overnight. The samples were embedded in paraffin and cut into 7-µm-thick sections.

### 2.6. Immunohistochemistry

Paraffin-embedded specimens were cut into 7-μm-thick sections and mounted on glass slides. After drying overnight at room temperature to allow specimens to adhere to slides, paraffin was dissolved in a slide heater (ThermoBrite; Leica Biosystems, Nussloch, Germany) at 65 °C for 30 min immediately before use. The slides were then deparaffinized by immersion in xylene twice at room temperature (5 min each). Slides were transferred twice to 100% ethanol (3 min each), once to 95%, 70%, and 50% ethanol (3 min each) and rehydrated at room temperature. Subsequently, 1.5% bovine serum albumin was used for blocking for 1 h at room temperature, and mouse primary antibodies were localized on mouse tissue using the Vector^®^ M.O.M. kit (Vector Laboratories Inc., Burlingame, CA, USA). After washing three times with PBS, slides were incubated with mouse monoclonal Shh antibody (Santa Cruz Biotechnology, Inc., Dallas, TX, USA). Subsequently, after washing thrice with PBS, slides were incubated with Alexa Fluor 488-conjugated goat anti-mouse antibody (1:200 in PBS; Thermo Fisher Scientific, Waltham, MA, USA) for 1 h at room temperature, washed three times with PBS, and then embedded in ProLong gold (Thermo Fisher Scientific). The slides were then observed under an all-in-one stereomicroscope (BZ-X800, KEYENCE, Osaka, Japan). The relative intensity of the fluorescent signal was quantified as arbitrary units (AU) by measuring the average fluorescent intensity of a 10-µm^2^ sized area at a minimum of 10 locations in the wound. Data from uninjured tissue were subtracted to remove nonspecific background fluorescence.

### 2.7. Cell Proliferation Assay

The PAM212 mouse keratinocyte cell line was obtained from Thermo Fisher Scientific. Briefly, 1 × 10^4^ cells were seeded on a 96-well plate and treated with 2% fetal bovine serum (FBS; control), 50 μg/mL recombinant Shh + 2% FBS, or 50 μg/mL cyclopamine + 2% FBS. Cells were incubated at 37 °C for 48 h, and cell proliferation was evaluated using the CellQuanti-MTT Cell Viability Assay Kit (BioAssay Systems, Hayward, CA, USA).

### 2.8. Scratch Assay

Briefly, 1 × 10^4^ PAM212 was seeded on a plastic dish and grown to confluence. Cells were scratched with a cell scraper with a width of 500 µm. Cells were cultured in Dulbecco’s Modified Eagle’s Medium (DMEM) supplemented with 50 μg/mL of recombinant Shh or cyclopamine (LKT Laboratories, Inc., St Paul, MN, USA) for 24 h. The edges of each scratch were examined under a microscope and the wound area was measured using ImageJ software (ver. 1.53p, National Institutes of Health, Bethesda, MD, USA). The experiment was independently repeated three times.

### 2.9. RNA Extraction and Reverse Transcription

The wounded skin was collected under a stereomicroscope by cutting as close to the wound margin as possible. A monophasic solution of phenol and guanidine isothiocyanate (ISOGEN; Nippon Gene, Tokyo, Japan) was used to extract total skin RNA from both injured and uninjured specimens according to the manufacturer’s instructions. The cDNA synthesis by reverse transcription was performed after mixing the extracted total RNA with random primers, reverse transcriptase, and dNTP mixture (Takara Bio Inc., Shiga, Japan).

### 2.10. Reverse Transcription Quantitative Polymerase Chain Reaction (RT-qPCR)

RT-qPCR and transcript quantification were performed on an Applied Biosystems 7500 Fast real-time PCR system (Thermo Fisher Scientific) using the TaqMan Gene Expression Master Mix (Thermo Fisher Scientific) in different samples (in triplicate) with the following primers for the quantification of transcript levels: Shh (Mm00436528_m1), patched1 (Mm00436026_m1), Gli1 (Mm00494654_m1), Gli2 (Mm01293116_m1), Gli3 (Mm00492337_m1), Cyclin D1 (Mm00432359_m1), Cyclin D2 (Mm00438070_m1). The housekeeping gene ACTB (Mm02619580_g1) was an endogenous normalization control. The level of gene expression in the proliferating cell population was a baseline and the fold change values were determined using the 2^-ΔΔCt^ method.

### 2.11. In Situ Hybridization

In situ hybridization analysis was performed using the QuantiGene ViewRNA ISH Tissue Assay (Thermo Fisher Scientific) according to the manufacturer’s instructions. Briefly, paraffin sections were dried at 60 °C for 60 min and paraffin removal was performed with Histo Clear (National Diagnostics, Atlanta, GA, USA) and 100% ethanol with an ImmEdge Pen. After washing twice with PBS, the tissue was fixed in 10% neutral buffered formaldehyde solution for 5 min and washed again with PBS. The target probe was diluted 50-fold in probe set diluent QF solution heated to 40 °C and incubated at 40 °C for 3 h. After washing three times with wash buffer, the probe was incubated in a preamplifier solution at 40 °C for 25 min. Following another wash with wash buffer three times, the probe was incubated in a preamplifier solution at 40 °C for 15 min. The AP enhancer solution was decanted and the Fast Red Tablet was dissolved in Napthol buffer and incubated at room temperature for 5 min. After decanting the AP enhancer solution, the Fast Red Tablet was dissolved in Napthol buffer and incubated at 40 °C for 30 min. After being washed twice with PBS, nuclear staining was performed with Gill’s Hematoxylin solution and washed three times with water. The probe used was Shh (VB6-13424-VC).

### 2.12. Statistical Analysis

Mann–Whitney *U* tests were performed to determine differences in migration or gene expression using Statistica software version 9.0 (StatSoft, Tulsa, OK, USA). All values are presented as mean ± SD. The threshold for statistical significance was established at *p <* 0.05. Each experiment was conducted in triplicate.

## 3. Results

### 3.1. Shh Expression Pattern in Mouse Wounds

**Figure 1:**
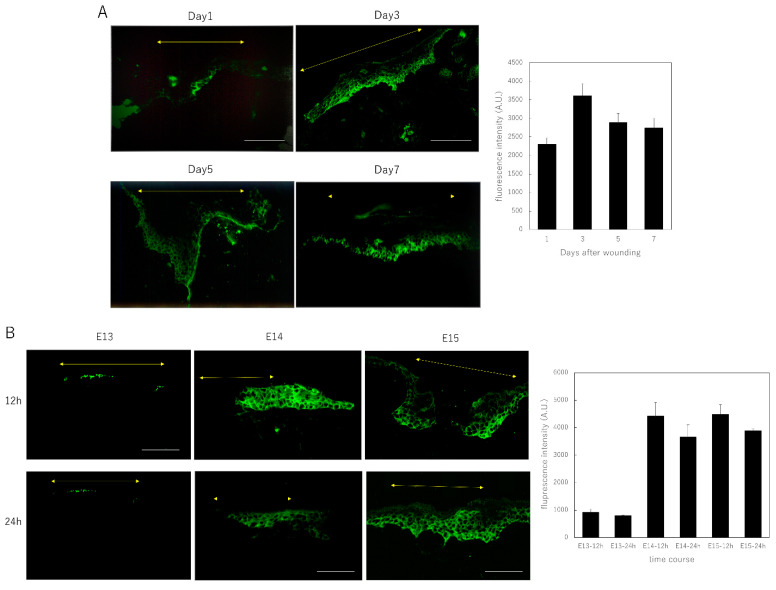

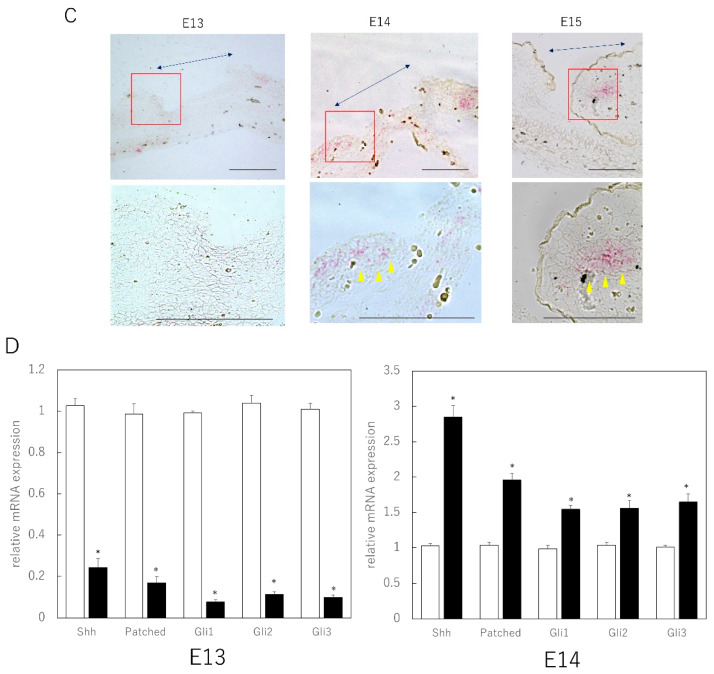
Expression of Shh during wound healing in mice. (**A**) Immunostaining analysis of Shh expression during wound healing in adult mice. The yellow arrows indicate the extent of the wound. Bar = 200 µm. To compare Shh expression, we quantified the average fluorescence intensity in at least 10 areas within the wound, in sections 10 µm^2^. (**B**) Immunostaining analysis of Shh expression during wound healing in fetal mice. Yellow arrows indicate the extent of the wound; Bar = 100 µm. To compare Shh expression, we quantified the average fluorescence intensity in at least 10 areas within the wound, in sections 10 µm^2^. (**C**) In situ hybridization analysis of the expression of Shh 24 h after fetal mouse wounding. The black arrows indicate the extent of the wound. The tip of the wound, circled with a red square, is magnified in the bottom row. The blue arrows indicate the area of the wound. Yellow arrows indicate the expression of Shh in the dermis. Bar = 100 µm. (**D**) Expression of Shh signaling-related genes in E13 and E14. The expression of Shh and all the signaling molecules involved in Shh signaling was decreased in E13 mouse wounds compared to normal skin, whereas the expression of Shh and the signaling molecules downstream of patched1 increased in wounds in E14. * *p <* 0.05.

First, we investigated the expression of Shh in mouse wounds during development from embryonic to postnatal stages. We found that Shh was expressed in the epidermis of adult mice at all timepoints, 1, 3, 5, and 7 d after wounding (Figure 1A), particularly in the inner layer of the thickened re-epithelialized epidermis at the wound margin. We specifically observed that the staining was present 24 h after wounding, became more intense on days 3–5, and persisted until the thickened epidermis thinned again on day 7. We also found that Shh was expressed in the epidermis of embryonic mice at the wound margin 12 h after wounding at E14 and E15, when the chymes no longer regenerated, but was barely expressed at E13 (Figure 1B). We also observed that Shh was not expressed in the wound at E13, whereas it was expressed in the epidermis from E14 onward, as revealed by in situ hybridization (Figure 1C).

Using RT-PCR, we detected that the expression of Shh and all signaling molecules involved in canonical Shh signaling, such as patched1, Gli1, Gli2, and Gli3, was downregulated in the wounds of E13 mice compared to normal skin. However, the expression of Shh and signaling molecules downstream of patched1 increased in the wound as the developmental stage progressed (Figure 1D).

### 3.2. Effect of Cyclopamine on Wound Healing in Adult Mice

**Figure 2:**
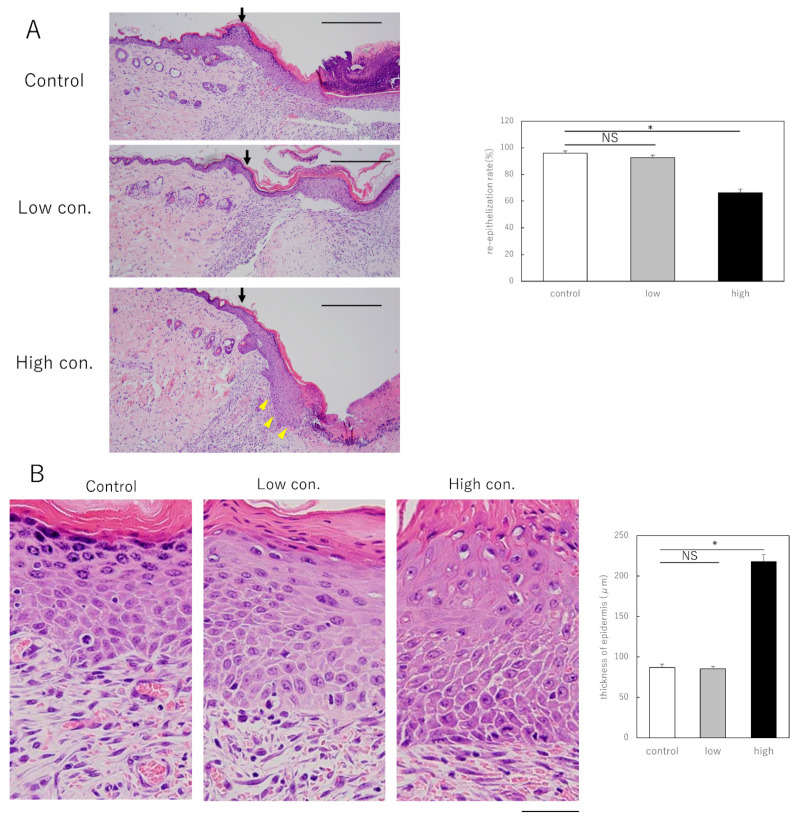
Effect of cyclopamine administration on wounds from adult mice. (**A**) Comparison of epithelialization rate after cyclopamine administration. Black arrows indicate the boundaries of the scar. Yellow arrows indicate thickened epidermis. Bar = 100 µm. * *p <* 0.05. (**B**) Comparison of epidermal thickness in the scar area after cyclopamine administration. Bar = 20 µm. * *p <* 0.05. NS; not significant. Cyclopamine at concentrations of at least 50 µg/µL caused prolonged epithelialization and epidermal thickening in adult mouse wounds.

**Figure 3:**
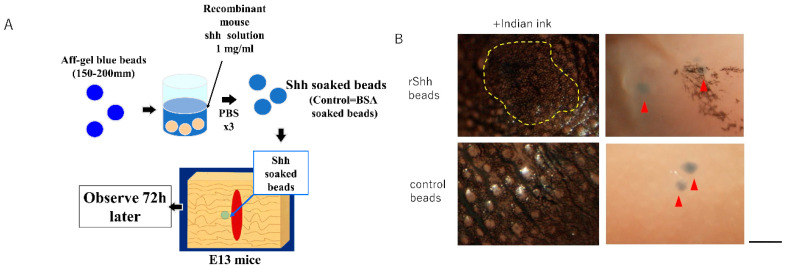
Transplantation of recombinant Shh-containing beads into E13 mice. (**A**) Schematic of the experiment. (**B**) Wound 72 h after bead implantation. Red arrows indicate the transplanted beads. Bar = 1 mm.

Next, we investigated the effect of suppressing Shh signaling on wound healing in adult mice. We administered cyclopamine, an inhibitor of the Hedgehog signaling pathway, to wounds of adult mice daily. To determine how cyclopamine affects wound healing in adult mice, two concentrations (low, 500 ng/µL or high, 50 µg/µL) were administered. Therefore, epithelialization was suppressed in mice treated with high concentrations (Figure 2A). Furthermore, focusing on the thickness of the epidermis at the margin of the wound, we observed a notable increase in stratification (Figure 3B). We found that cyclopamine administration at least 50 µg/µL causes prolonged epithelialization and epidermal thickening via inhibition of Shh.

### 3.3. Disturbance of Skin Texture Pattern Due to Recombinant Shh

To investigate the possibility that Shh signaling might be involved in regeneration of skin texture regeneration, we implanted slow-release beads soaked in recombinant Shh in the wounds of E13 mice and expected that they should completely regenerate skin texture (Figure 2A). We found that the application of beads compensated for the reduced expression of Shh in the wounds of E13 mice and mimicked the Shh-expressing wounds from E14 onward. Interestingly, we observed that the bead-containing wounds showed a loss of texture pattern 72 h after injury (Figure 2B).

### 3.4. The Expression of Shh Affected Epidermal Cell Proliferation and Migration Capacity

**Figure 4:**
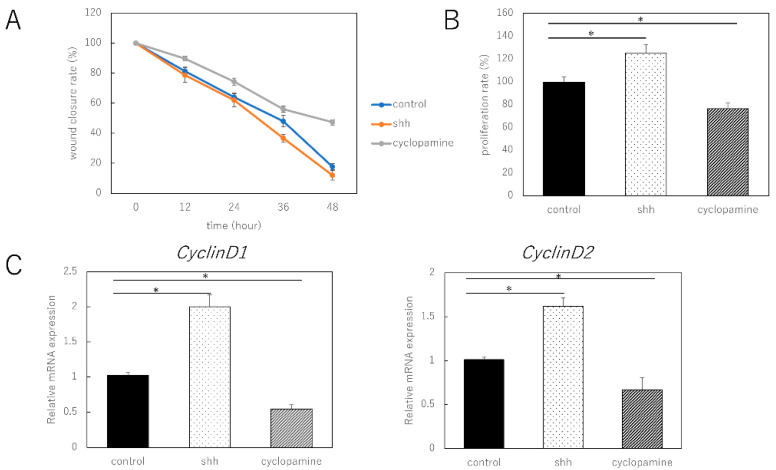
Effect of Shh on epidermal cells in vitro. (**A**) Comparison of migration ability using a scratch assay. (**B**) Comparison of the cell proliferation rate. * *p <* 0.05. (**C**) Comparison of the expression of cyclin D genes. * *p <* 0.05.

To investigate the mechanism of the epidermal thickening observed in vivo, we examined the proliferative and migratory capacity of epidermal cells in the presence of Shh inhibitors and recombinant Shh. We found that recombinant Shh promoted the proliferation of epidermal cells, whereas cyclopamine treatment inhibited their proliferation (Figure 4A). Furthermore, we detected that recombinant Shh promoted cell migration in the scratch assay, whereas cyclopamine inhibited it (Figure 4B). Moreover, the expression of the cyclin D1 and D2 genes was decreased in epidermal cells under Shh inhibition, whereas upregulated after treatment with recombinant Shh (Figure 4C).

## 4. Discussion

Epidermal–dermal interactions and patterning factors might be involved in the processes leading to loss of complex skin texture during wound healing; however, the underlying mechanisms of these regenerative processes have not been elucidated. We observed the expression of Shh, a factor involved in patterning and hair follicle formation, during the development of fetal mouse and in wound healing in adult animals. We also examined the effects of its expression on skin texture formation.

Although Shh signaling is closely involved in hair follicle development and skin tumorigenesis, the underlying mechanisms and downstream events it coordinates on the skin remain elusive. Here, we observed that Shh signaling was downregulated in the wounds of E13 mice with regenerating skin texture, and external Shh supplementation inhibited texture regeneration.

In our previous study, we reported that complete skin regeneration, including skin texture, requires epidermal and dermal positioning [1]. At E13, the wound contracts with the epidermis always in contact with the dermis during the wound-healing process, but after E14, the epidermis migrates faster than the dermis and the epidermal-dermal interaction is lost when the epidermis tip contacts the fascia, resulting in the presence of visible marks [12]. In this study, the expression of Shh signaling was downregulated in wounds at E13, whereas cell migration and proliferation were enhanced in the presence of Shh in vitro, suggesting that Shh controls epidermal migration to maintain its positional relationship with the dermis.

Shh plays an important role in both fetal and adult hair development, and in mice lacking Shh, mature hair follicles do not develop, despite the initiation of follicle formation and the formation of dermal condensates [13,14,15]. Furthermore, treatment with Shh inhibitory antibodies has been shown to cause reversible alopecia due to the arrest of hair follicles in the resting phase, which indicates that Shh is also required for the postnatal skin hair cycle [16]. These findings have suggested that Shh is necessary in the formation of hair follicles; however, dermal aggregation and placode formation occur in the developmental stages after E14, consistent with the downregulation of Shh at E13.

In various developmental situations, Hedgehog signaling is associated with proliferative responses of target cells. For example, quiescent cells are stimulated to enter the cell cycle in response to mitogenic signals such as Shh that induce the expression of cyclin D proteins, which is required to pass the G1 restriction point [17]. Shh signaling, which is required for chondrocyte proliferation, also regulates cyclin D1 expression in developing bone [18]. The expression of cyclin D proteins is strictly regulated at both the transcriptional and posttranscriptional levels; however, it has also been reported that the expression of cyclin D1 is controlled by Shh-dependent and Shh-independent signaling in embryonic skin [19,20]. Furthermore, cyclin D1 and D2 proteins are downregulated in *Shh* and *Gli2* mutant skin and are induced by the activator function of Gli2 [11], no embryonic skin phenotype has been reported for either cyclin D1 -/- or cyclin D2 -/- mutant mice. Our results suggested that the downregulation of Shh in E13 skin and the Shh-dependent changes in cyclin D observed in vitro were responsible for this cell proliferation signal.

We also provided evidence that the Shh pathway might regulate keratinocyte motility. In a previous report, overexpression of Shh induced the invasion of HaCaT cells, which depended on an intact EGF signaling axis, as opposed to the inhibition of neural crest cell motility [21]. Although the response elicited by Shh might be cell context dependent, it is reasonable to speculate that the Shh pathway might play an important role in keratinocyte adhesion and motility during wound healing.

The Hedgehog pathway includes canonical signaling mediated by Smoothened (Smo) activation and induces stabilization and activation of the Gli family of potential zinc finger transcription factors; in contrast, there is non-canonical signaling only via patched, which functions as a dependent receptor independent of Smo through regulation of cyclin B1 and caspase-9 [22]. The Shh signaling identified in Drosophila is consistent with the canonical pathway and is involved in cell migration and proliferation [23]. In this study, we show that Shh expression in fetal mouse wounds determines regeneration and non-regeneration via changes in patched and Gli family expression, and that at least the classical pathway of Shh affects the wound-healing phenotype. However, the possibility of influence through non-classical pathways will need to be pursued in the future.

A limitation of our study was that we found a contradiction in that downregulation of Shh signaling is necessary for skin texture regeneration but detrimental to hair follicle regeneration. Thus, simply downregulating Shh to achieve a wound-healing scheme that regenerates the skin, including its texture, as in E13, leads to inhibition of hair follicle formation. Therefore, it will be necessary to create mice with epidermal cell-specific conditional knockout of Shh or knockout of downstream targets of Shh signaling—cyclin D and Gli family members, and observe the phenotypic changes in skin texture.

In this study, we showed that Shh signaling was altered during the healing of fetal mouse wounds at E13 and E14 and beyond, specified the transition point for texture regeneration, and revealed that Shh affected the motility and proliferative capacity of epidermal keratinocytes. These findings could contribute to the development of methods for the regeneration of skin texture and the healing of scars without a visible mark.

## Data Availability

The data presented in this study are available on request from the corresponding author.

## References

[1] Takaya K., Okabe K., Ishigami A., Imbe Y., Kanazawa H., Sakai S., Aramaki-Hattori N., Kishi K. (2022). Actin Cable Formation and Epidermis–Dermis Positional Relationship during Complete Skin Regeneration. Sci. Rep..

[2] Armstrong J.R., Ferguson M.W. (1995). Ontogeny of the skin and the transition from scar-free to scarring phenotype during wound healing in the pouch young of a marsupial, Monodelphis domestica. Dev. Biol..

[3] Ishii T., Takashimizu I., Casco-Robles M.M., Taya Y., Yuzuriha S., Toyama F., Maruo F., Kishi K., Chiba C. (2021). Skin wound healing of the adult newt, Cynops pyrrhogaster: A unique re-epithelialization and scarless model. Biomedicines.

[4] Weasner B.M., Kumar J.P. (2022). The timing of cell fate decisions is crucial for initiating pattern formation in the Drosophila eye. Development.

[5] Zuniga A. (2015). Next generation limb development and evolution: Old questions, new perspectives. Development.

[6] Lim C.H., Sun Q., Ratti K., Lee S.H., Zheng Y., Takeo M., Lee W., Rabbani P., Plikus M.V., Cain J.E. (2018). Hedgehog stimulates hair follicle neogenesis by creating inductive dermis during murine skin wound healing. Nat. Commun..

[7] Widelitz R.B., Chuong C.M. (1999). Early events in skin appendage formation: Induction of epithelial placodes and condensation of dermal mesenchyme. J. Investig. Dermatol. Symp. Proc..

[8] Sengel P. (1990). Pattern formation in skin development. Int. J. Dev. Biol..

[9] Rognoni E., Gomez C., Pisco A.O., Rawlins E.L., Simons B.D., Watt F.M., Driskell R.R. (2016). Inhibition of β-catenin signalling in dermal fibroblasts enhances hair follicle regeneration during wound healing. Development.

[10] Hamburg-Shields E., DiNuoscio G.J., Mullin N.K., Lafyatis R., Atit R.P. (2015). Sustained β-catenin activity in dermal fibroblasts promotes fibrosis by up-regulating expression of extracellular matrix protein-coding genes. J. Pathol..

[11] Wier E.M., Garza L.A. (2020). Through the lens of hair follicle neogenesis, a new focus on mechanisms of skin regeneration after wounding. Semin. Cell Dev. Biol..

[12] Wang Y., Guerrero-Juarez C.F., Qiu Y., Du H., Chen W., Figueroa S., Plikus M.V., Nie Q. (2019). A multiscale hybrid mathematical model of epidermal-dermal interactions during skin wound healing. Exp. Dermatol..

[13] St-Jacques B., Dassule H.R., Karavanova I., Botchkarev V.A., Li J., Danielian P.S., McMahon J.A., Lewis P.M., Paus R., McMahon A.P. (1998). Sonic hedgehog signaling is essential for hair development. Curr Biol..

[14] Mill P., Mo R., Fu H., Grachtchouk M., Kim P.C., Dlugosz A.A., Hui C.C. (2003). Sonic hedgehog-dependent activation of Gli2 is essential for embryonic hair follicle development. Genes Dev..

[15] Karlsson L., Bondjers C., Betsholtz C. (1999). Roles for PDGF-A and sonic hedgehog in development of mesenchymal components of the hair follicle. Development.

[16] Wang L.C., Liu Z.Y., Gambardella L., Delacour A., Shapiro R., Yang J., Sizing I., Rayhorn P., Garber E.A., Benjamin C.D. (2000). Regular articles: Conditional disruption of Hedgehog signaling pathway defines its critical role in hair development and regeneration. J. Investig. Dermatol..

[17] Kenney A.M., Rowitch D.H. (2000). Sonic hedgehog promotes G(1) cyclin expression and sustained cell cycle progression in mammalian neuronal precursors. Mol. Cell Biol..

[18] Long F., Zhang X.M., Karp S., Yang Y., McMahon A.P. (2001). Genetic manipulation of hedgehog signaling in the endochondral skeleton reveals a direct role in the regulation of chondrocyte proliferation. Development.

[19] Fantl V., Stamp G., Andrews A., Rosewell I., Dickson C. (1995). Mice lacking cyclin D1 are small and show defects in eye and mammary gland development. Genes Dev..

[20] Sicinski P., Donaher J.L., Geng Y., Parker S.B., Gardner H., Park M.Y., Robker R.L., Richards J.S., McGinnis L.K., Biggers J.D. (1996). Cyclin D2 is an FSH-responsive gene involved in gonadal cell proliferation and oncogenesis. Nature.

[21] Bigelow R.L., Jen E.Y., Delehedde M., Chari N.S., McDonnell T.J. (2005). Sonic hedgehog induces epidermal growth factor dependent matrix infiltration in HaCaT keratinocytes. J. Investig. Dermatol..

[22] Teperino R., Aberger F., Esterbauer H., Riobo N., Pospisilik J.A. (2014). Canonical and Non-Canonical Hedgehog Signalling and the Control of Metabolism. Semin. Cell Dev. Biol..

[23] Araújo S. (2015). The Hedgehog Signalling Pathway in Cell Migration and Guidance: What We Have Learned from Drosophila Mel anogaster. Cancers.

